# *TLR3* rs3775291 C/T polymorphism is associated with elevated IFN-α level in asymptomatic HTLV-1 infection

**DOI:** 10.3389/fcimb.2025.1604259

**Published:** 2025-08-04

**Authors:** Fabiane dos Santos Ferreira, Wandrey Roberto dos Santos Brito, Emmanuelle Giuliana Mendes Santana, Aline Kelly Alves Oliveira, Aline Cecy Rocha de Lima, Ednelza da Silva Graça Amoras, Carlos Araujo da Costa, Maísa Silva Souza, Sandra Souza Lima, Ricardo Ishak, Izaura Maria Vieira Cayres-Vallinoto, Antonio Carlos Rosário Vallinoto, Maria Alice Freitas Queiroz

**Affiliations:** ^1^ Laboratory of Virology, Institute of Biological Sciences, Federal University of Pará, Belém, Brazil; ^2^ Graduate Program in Biology of Infectious and Parasitic Agents, Institute of Biological Sciences, Federal University of Pará, Belém, Brazil; ^3^ Laboratory of Cellular and Molecular Biology, Tropical Medicine Center, Federal University of Pará, Belém, Brazil

**Keywords:** HTLV-I infection, inflammatory diseases, toll-like receptor 3, polymorphism genetic, cytokines

## Abstract

**Introduction:**

Alterations in the immune response may influence the development of HTLV-1-associated diseases. TLR3 detects viral nucleic acids, including HTLV-1, and triggers the production of IFN-I and other cytokines. Genetic variations in *TLR3* may alter the antiviral and inflammatory responses and contribute to the progression of HTLV-1 infection. The present study investigated the association of polymorphisms in the *TLR3* gene (rs5743305 T/A and rs3775291 C/T) with HTLV-1 infection status and their relationship with infection-associated diseases, receptor expression levels, proviral load, and inflammatory and antiviral cytokines.

**Methods:**

Blood samples were collected from 179 individuals with HTLV-1 infection (82 with inflammatory diseases and 97 asymptomatic individuals) and 179 controls. Genotyping of polymorphisms, analysis of *TLR3* gene expression, and quantification of proviral load were performed by real-time PCR, and cytokine levels were measured by enzyme-linked immunosorbent assay (ELISA).

**Results:**

The polymorphisms showed no correlation with susceptibility to HTLV-1 infection, or the occurrence of disease symptoms linked to the infection. The presence of disease symptoms was associated with higher TNF-α levels and proviral load. *TLR3* rs5743305 T/A polymorphism was not associated with variations in *TLR3* and IFN-α levels. For the *TLR3* rs3775291 C/T polymorphism, asymptomatic individuals carrying the TT polymorphic genotype presented significantly higher IFN-α levels and lower proviral load. The profile of asymptomatic individuals carrying the polymorphic genotypes for *TLR3* rs3775291 C/T was characterized by higher levels of *TLR3* and IFN-α and lower levels of proviral load, TNF-α and IL-6 compared to those with the wild-type genotype.

**Conclusion:**

Although polymorphisms in the TLR3 gene have not been associated with the presence of symptoms of HTLV-1-related inflammatory diseases, the *TLR3* rs3775291 C/T polymorphism appears to contribute to a better evolution of the HTLV-1 infection status and inflammatory process among asymptomatic individuals.

## 1 Introduction

The majority of human T-lymphotropic virus 1 (HTLV-1) infections are asymptomatic. However, 5 to 10% of infected individuals may develop different types of diseases, including adult T-cell leukemia (ATL) and inflammatory diseases such as HTLV-1-associated myelopathy (HAM), which causes demyelination of neurons, as well as dermatitis, uveitis, and rheumatic diseases (Schierhout et al., 2020; Kalyanaraman et al., 1982; Osame et al., 1986; Kamoi and Mochizuki, 2012). Factors that may contribute to the development of diseases associated with HTLV-1 are being investigated, such as high levels of proviral load (Nagai et al., 1998; Taylor et al., 1999; Pineda et al., 2019) and changes in the immune response against the virus, which are associated with an increased risk of progression to diseases (Yamauchi et al., 2021; Brites et al., 2021; Clauze et al., 2022).

Genetic variations in components of the immune response have been the focus of several studies in the search for biomarkers that may justify differences in the evolution of infection among individuals with HTLV-1, since some people develop pathological conditions, while others remain in an asymptomatic state (Queiroz et al., 2020; Rafatpanah et al., 2021; Santana et al., 2024). Among the various components of innate immunity, Toll-like receptors (TLRs) stand out for their diversity and for initiating the activation of one of the main intracellular signaling pathways, responsible for promoting an antiviral and inflammatory state during various types of viral infections (Akira and Takeda, 2004). The TLR3 receptor, located in the endosome, detects double-stranded RNA (dsRNA). dsRNA is characterized as a byproduct resulting from the replication and transcription process of several viruses, including HTLV (Van montfoort et al., 2014; Mohanty and Harhaj, 2023).

TLR3 activation results in the synthesis of type I IFNs (IFN-I) and pro-inflammatory cytokines (Vercammen et al., 2008; Mcgettrick and O’neill, 2010). FN-I (IFN-α and IFN-β) have potent antiviral properties, as they interfere with virus replication and act in the immunomodulation of adaptive immunity (Biron, 2001; Xagorari and Chlichlia, 2008; Uematsu and Akira, 2007; Carty et al., 2021). Kinpara et al. (2009) showed that both IFN-α and IFN-β are capable of directly suppressing HTLV-1 expression in HTLV-1-infected cell lines and primary ATL cells (Kinpara et al., 2009).

Alterations in the structure or function of TLR3 may play a crucial role in the progression of viral infections and the potential development of symptoms associated with different clinical conditions linked to HTLV-1. The *TLR3* gene is located on chromosome 4 (4q35.1), where some polymorphisms are found (Fischer et al., 2018; Habibabadi et al., 2020; Redondo et al., 2022). The *TLR3* rs3775291 C/T genetic variant is located in exon four of the *TLR3* gene, generating an amino acid change from leucine to phenylalanine at position 412 of the protein (L412F) (Ranjith-kumar et al., 2007). This alteration may change the binding capacity of dsRNA and influence intracellular signaling when compared to the wild type (Ranjith-kumar et al., 2007; Brown and Razonable, 2010; Zhou et al., 2011). The presence of the rs3775291 C/T polymorphism in the *TLR3* gene can generate functional alterations and impact the activation of TLR3-mediated immune responses, influencing the intensity of the inflammatory response. The *TLR3* rs3775291 C/T polymorphism has been studied in relation to different viral infections, such as HIV-1 (Sironi et al., 2012), cytomegalovirus (Redondo et al., 2022), tick-borne encephalitis (Kindberg et al., 2011) and hepatitis B virus (HBV) (Chen et al., 2017).

The *TLR3* rs5743305 T/A polymorphism is situated in the promoter region of the *TLR3* gene and can affect its transcriptional activity, thereby modifying gene expression compared to the wild type. Alteration in TLR3 transcriptional activity may influence receptor functionality and the development of viral diseases. The TLR3 rs5743305 T/A polymorphism has been studied in relation to various viral infections, including HBV (Fischer et al., 2018), hepatitis C virus (HCV) (Askar et al., 2009), and tick-borne encephalitis (Grygorczuk et al., 2017).

In HTLV-1 infection, a single study examined polymorphisms in the *TLR3* gene, identifying *TLR3* rs3775296 as a potential protective factor against infection (Habibabadi et al., 2020). The absence of comprehensive studies analyzing polymorphisms in the *TLR3* gene constitutes a significant limitation in understanding the impact of genetic variations in this receptor on HTLV-1 infection. The present study investigated the association of polymorphisms in the *TLR3* gene (rs5743305 T/A and rs3775291 C/T) with HTLV-1 infection and infection status (related to the presence or absence of symptoms of inflammatory disease). The influence of polymorphisms in the *TLR3* gene on the levels of proviral load and inflammatory and antiviral cytokines according to the HTLV-1 infection status was also analyzed. The *TLR3* rs5743305 T/A and *TLR3* rs3775291 C/T polymorphisms were selected for this study because they had not yet been evaluated in HTLV-1 infection, although they were associated with other viral infections.

## 2 Materials and methods

### 2.1 Study characterization

This cross-sectional study included 179 individuals infected with HTLV-1. Among them, 82 patients had a clinical diagnosis of inflammatory diseases such as HAM, rheumatological manifestations, dermatitis, pulmonary complications, and uveitis, while 97 individuals were asymptomatic. All individuals infected with HTLV-1 were categorized as people living with HTLV-1. The symptomatic group consisted of those who had a clinical diagnosis of any inflammatory disease assessed in the study. Conversely, the asymptomatic group comprised individuals with HTLV-1 who exhibited no clinical symptoms of disease. The primary clinical symptoms observed in HAM patients included lower back pain, urinary urgency, erectile dysfunction, fatigue, falls, leg pain, changes in gait, with one patient requiring a wheelchair.

Specialist physicians conducted the clinical diagnosis of inflammatory diseases linked to HTLV-1 according to a standard protocol. No patients with HTLV-2 were included in the study. Individuals of both sexes and over 18 years old, who were treated at the outpatient clinic of the Center for Tropical Medicine and the Service for Assistance to People Living with HTLV (SAPEVH) at the Federal University of Pará, were included. The samples were collected in Belém, Pará, Northern region of Brazil, from January 2023 to November 2024.

In Brazil, the highest infection rates are observed in the North and Northeast regions of the country (Legrand et al., 2022). Our study focuses on the population of the city of Belém, located in the state of Pará, which is within the North region of Brazil. HTLV-1/2 infections are endemic to the state of Pará (Silva et al., 2018; Lopes et al., 2022).

Individuals receiving glucocorticoid treatment, those co-infected with HTLV-2, HIV-1/2, HCV and HBV, and those with autoimmune disease were excluded from the study.

A sample containing 10 mL of blood was collected by intravenous puncture using a vacuum collection system containing ethylenediaminetetraacetic acid (EDTA) as an anticoagulant. The samples were centrifuged and separated into aliquots of plasma and leukocytes. An aliquot of the leukocyte samples was used for genomic DNA extraction to perform genotyping of polymorphisms in the *TLR3* gene (rs5743305 T/A and rs3775291 C/T) and to quantify proviral load. Another aliquot of the leukocyte samples was used for RNA extraction to quantify *TLR3* gene expression. Plasma samples were used to measure plasma cytokines.

A comparison cohort was constituted from 179 blood samples provided by individuals who voluntarily sought testing for HTLV-1 infection at SAPEVH and received a negative diagnosis. All participants were over 18 years of age. Individuals with other infections, including HTLV-2, HIV-1/2, HCV, and HBV, as well as those with autoimmune diseases, were excluded from the cohort. The individuals were selected from Jan/2023 to Nov/2024. The control group samples were utilized to compare the frequencies of polymorphisms, as they represented the same population group as the individuals with HTLV-1. Consequently, there were no interethnic differences observed. Control group samples were matched in age (mean: 49.5; median: 50; IIQ: 22) and sex with the HTLV-1 group.

### 2.2 DNA extraction

DNA extraction from whole blood leukocytes used the Puregene™ kit (Gentra Systems, Inc., Minneapolis, Minnesota, USA), following the manufacturer’s protocol, which consisted of the steps of cell lysis, protein precipitation, DNA precipitation and hydration. After extraction, the DNA obtained was quantified by spectrophotometric reading on the BioDrop™ equipment (Bio-Rad, Hercules, California, USA), following the protocol recommended by the manufacturer.

### 2.3 Genotyping of *TLR3* rs5743305 T/A and *TLR3* rs3775291 C/T

The genotypes of the investigated polymorphisms were identified by the real-time PCR technique, using the StepOnePLUS™ Real-Time PCR System (Thermo Fisher, Carlsbad, Califórnia, EUA). Commercial assays containing TaqMan™ hydrolysis probes with the identification C::393058_10 and C:_1731425_10 were used for genotyping the *TLR3* rs5743305 and *TLR3* rs3775291 polymorphisms, respectively (Thermo Fisher, Carlsbad, California, USA). These assays contained specific primers and probes for amplification of the target sequence. For each reaction, Mastermix (1x) (Thermo Fisher, Carlsbad, California, USA), H2O, assay C::393058_10 or C:_1731425_10 (20X), and DNA (50 ng) were used. The following cycling conditions were used: 10 minutes at 95°C and 40 cycles of 15 seconds at 95°C and 1 minute at 60°C.

### 2.4 RNA extraction

Total RNA was extracted from peripheral blood leukocytes using the TRIzol™ Plus RNA Purification Kit (Thermo Fisher Scientific, Waltham, Massachusetts, USA). The steps followed the protocol recommended by the manufacturer. The concentration of the extracted RNA was determined using a BioDrop™ (Bio-Rad, Hercules, California, USA) according to the manufacturer’s instructions. All total RNA samples had concentrations equal to 50 ng/µL for the synthesis of complementary DNA (cDNA).

### 2.5 Reverse transcription

The synthesis of cDNA from RNA was performed using the High-Capacity cDNA Reverse Transcription^®^ with RNAse Inhibitor Kit (Applied Biosystems, Foster City, CA, USA). For each reaction, a mixture with a final volume of 20.0 µL was prepared, contained 2 µL of 10X RT Buffer, 0.8 µL of 25X dNTP Mix (100 nM), 2 µL of random primer, 1 µL of MultiScribe™ Reverse Transcriptase, 1 µL of RNaseOUT™ and 3.2 µL of ultrapure water supplied in the kit and 10.0 µL of extracted RNA. Subsequently, the mixture was placed in a Mastercycler personal thermal cycler (Eppendorf, Hamburg, Germany) and subjected to cycles of 25°C for 10 minutes, 37°C for 120 minutes and 85°C for 5 minutes.

### 2.6 Quantification of gene expression

Gene expression was measured by quantifying mRNA using real-time PCR. First, the standardization of the quantitative PCRs (qPCRs) with the cDNAs and probes (endogenous and target genes) was performed to calculate the efficiency of the amplification reactions. In the standardization reactions, different concentrations of cDNA were tested (pure and in 4 dilutions with a factor of 2 - 1:2, 1:4, 1:8 and 1:16). All reactions were conducted on plates and performed in triplicate. The same cDNA, at various dilutions, was analyzed with different probes to construct an efficiency curve and validate the 2-ΔΔCt analysis method. All tests showed efficiency as expected (100% ± 10) (Livak and Schmittgen, 2001).

The relative quantification of gene expression consisted of the amplification of the target gene with the endogenous gene (normalizer) using TaqMan™ assays (Applied Biosystems, Foster City, CA, USA) and the StepOnePLUS™ Real-Time PCR System (Thermo Fisher Scientific, Waltham, MA, USA). The reactions were performed in the singleplex format according to the manufacturer’s protocol. The assay used for *TLR3* was Hs01551078_m1, and glyceraldehyde-3-phosphate dehydrogenase (GAPDH) was used as the endogenous control (Hs02786624_g1). All assays were obtained commercially (Thermo Fisher Scientific, Waltham, MA, USA). For the reaction, 5 µL of 2X TaqMan^®^ Universal PCR Master Mix, 0.5 µL of 20X TaqMan Gene Expression Assay, 1 µL of cDNA and 10.5 µL of RNAse-free water were used, along with the following thermocycling conditions: 2 minutes at 50°C, followed by 10 minutes at 95°C and 1 minute at 60°C.

The relative quantification (RQ) of target gene expression was determined using the comparative CT method (ΔΔCt) with the formula 2-ΔΔCT, where ΔΔCt = ΔCt sample- ΔCt reference (Life Technologies, Carlsbad, CA, USA).

### 2.7 Plasma measurement of cytokine levels

The quantification of cytokines FN-α, IL-6, and TNF-α in plasma was conducted utilizing the Read y-SET-Go^®^ enzyme-linked immunosorbent assay (ELISA, eBioscience, California, San Diego, USA), which uses specific monoclonal antibodies to detect each cytokine and measure its level. The test was performed according to the manufacturer’s instructions.

### 2.8 Quantification of the HTLV-1 proviral load

The proviral load was quantified by qPCR using a TaqMan^®^ system (Applied Biosystems, Foster City, CA) with two target sequences (the albumin gene as an endogenous control and the nonhomologous region of the HTLV-1 pol gene), according to a previously described protocol (Tamegão-Lopes et al., 2006), which starts with the extraction of DNA from leukocytes, followed by relative quantification via real-time PCR. The result is further adjusted to an absolute proviral quantification considering the leukocyte count per mm^3^, and the result is expressed as the number of proviral DNA copies/mm^3^.

### 2.9 Statistical analysis

The genotypic and allelic frequencies of the polymorphisms were estimated by direct counting, and the significance of the differences between the studied groups was calculated using the chi-square (χ^2^) test and the G test. The Hardy-Weinberg equilibrium calculation was performed to evaluate whether the observed genotypic frequency distributions were in accordance with expectations. Analyses of the normality of the distribution of *TLR3* expression levels, the proviral load and cytokine levels were performed using the Shapiro−Wilk test. The *TLR3* expression levels, proviral load levels and cytokine levels among the investigated groups were evaluated using the nonparametric Mann−Whitney test. The multiple logistic regression test evaluated the associations of the investigated variables with the presence of disease symptoms. Multiple linear regression analysis was performed to evaluate the association between the gene expression of INF-α in relation to the proviral load of the virus, expression of *TLR3*, TNF-α, IL-6, *TLR3* rs5743305 polymorphism and *TLR3* rs3775291 polymorphism. The significance level adopted was 0.05. All tests will be performed using the R program and GraphPad Prism 5.0.

### 2.10 Ethical aspects

This study was submitted to and approved by the Ethics Committee for Research with Human Beings of the Institute of Health Sciences of the Federal University of Pará (CAAE no. 63427822.3.0000.0018). All individuals included in the study signed an informed consent form.

## 3 Results

Among the 179 individuals diagnosed with HTLV-1 who were evaluated in the study, 126 (70.3%) were female, with a mean age of 49.9 years (19–80 years; median: 50; IQR: 20). Most participants were female: 73.1% (n=60) in the symptomatic group and 67% (n=66) in the asymptomatic group (*p*= 0.2549). The mean age of the symptomatic group was 52.1 years (median 53.5; IQR: 19.2) and that of the asymptomatic group was 48.8 years (median: 49; IQR: 20).

### 3.1 Genotypic and allele frequency of *TLR3* rs5743305 T/A and *TLR3* rs3775291 C/T

The evaluation of genotypic and allelic frequencies for the *TLR3* rs5743305 T/A and *TLR3* rs3775291 C/T variations between the group of people living with HTLV-1 and the control group showed no statistically significant difference (**Table 1**). However, the comparison of genotypic and allelic frequencies for *TLR3* rs3775291 C/T among individuals with HTLV-1 according to the presence and absence of symptoms associated with infection showed that asymptomatic individuals had a higher frequency of the polymorphic genotype (*p*= 0.0206; *OR*= 0.24) and allele (*p*= 0.0044; *OR*= 0.48) (TT and T, respectively; **Table 2**).

**Table 1:** Comparison of the genotypic and allelic frequency of polymorphisms in the TLR3 gene between the groups with HTLV-1 and control.

Genotypic and allelic profile	HTLV-1 n= 179 n (%)	Control n= 179 n (%)	*p^x^*
*TLR3* rs5743305 T/A-926			
TT	96 (53.63)	88 (49.16)	0.6770
AT	70 (39.10)	78 (43.57)	
AA	13 (7.30)	13 (7.30)	
*T	0.73	0.71	0.5599
*A	0.27	0.29	
*TLR3* rs3775291 C/T1234			
CC	100 (55.87)	80 (44.69)	0.1068
CT	61 (34.08)	76 (42.46)	
TT	18 (10.05)	23 (12.85)	
*C	0.73	0.66	0.0516
*T	0.27	0.34	

n, number of individuals; *allele; ^x^chi-square test.

**Table 2:** Comparison of the genotypic and allelic frequency of polymorphisms in the *TLR3* gene between individuals with the presence and absence of symptoms of diseases associated with HTLV-1 infection.

Genotypic and allelic profile	Symptomatic n= 82 n (%)	Asymptomatic n= 97 n (%)	*p*	*OR* (IC 95%)
*TLR3* rs5743305 T/A-926				
TT	47 (57.32)	49 (50.51)	0.2126^g^	
AT	27 (32.93)	43 (44.34)		
AA	8 (9.75)	5 (5.15)		
*T	0.74	0.73	0.9089^x^	
*A	0.26	0.27		
*TLR3* rs3775291 C/T1234				
CC	54 (65.85)	46 (47.43)	0.0206^g^	0.24^a^ (0.07-0.79)
CT	24 (29.27)	37 (38.14)		
TT	4 (4.88)	14 (14.43)		
*C	0.80	0.66	0.0044^x^	0.48 (0.29-0.78)
*T	0.20	0.34		

n, number of individuals; *allele; ^g^teste G; ^x^teste qui-quadrado; ^a^TT *vs*. CC.

### 3.2 *TLR3* gene expression, plasma cytokines, and HTLV-1 proviral load among symptomatic and asymptomatic individuals for infection-associated diseases

**Figure 1:**
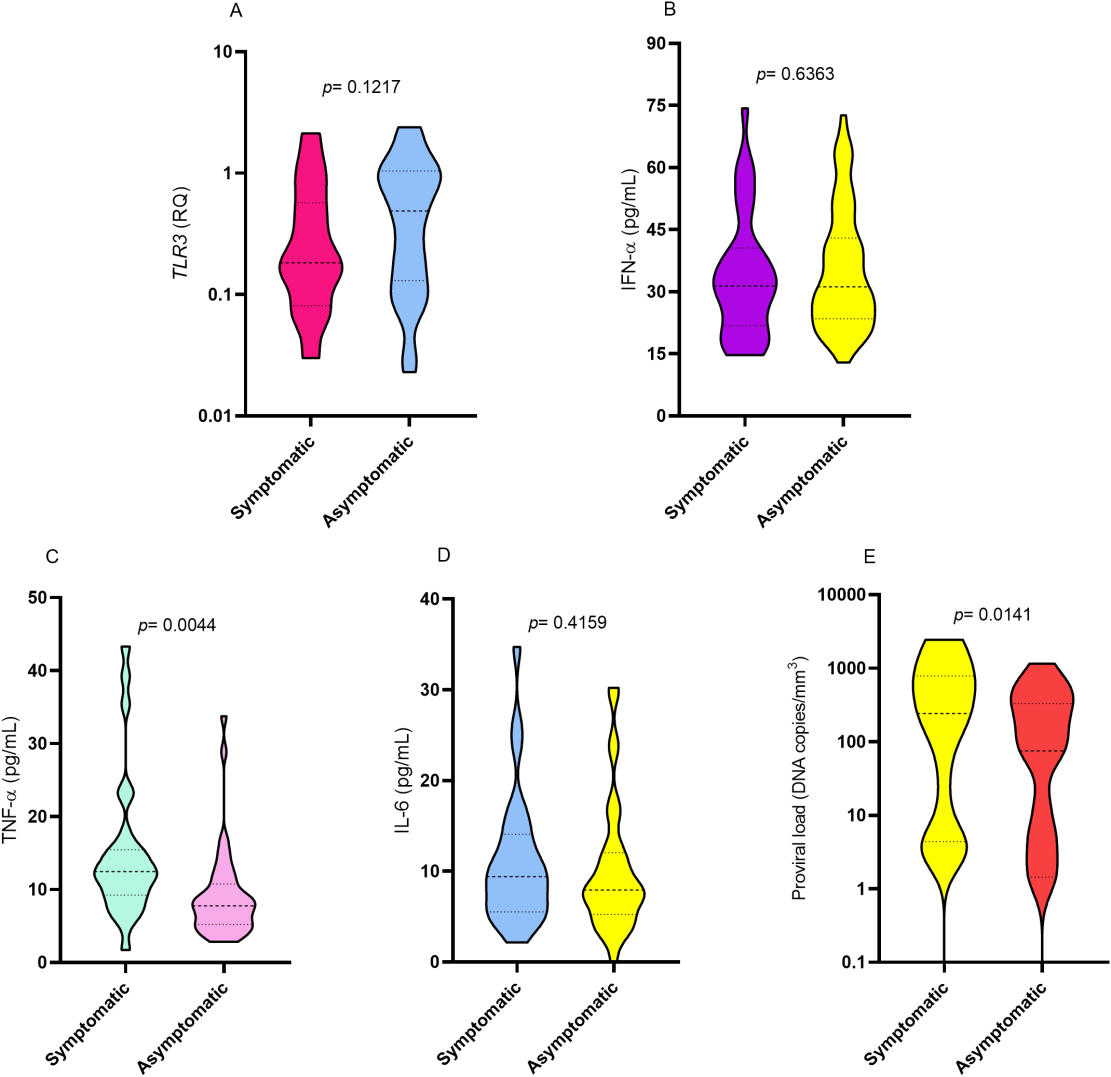
Comparison of the levels of **(A)** TLR3 gene expression, **(B)** IFN-α, **(C)** TNF-α, **(D)** IL-6 and **(E)** proviral load between symptomatic and asymptomatic individuals for HTLV-1-associated diseases. Mann-Whitney test.

The levels of *TLR3* expression, IFN-α, and IL-6 were not statistically different between the group with symptoms of HTLV-1-associated diseases and the group of asymptomatic individuals (p> 0.05; **Figures 1A, B, D**). However, plasma TNF-α (p= 0.0044; **Figure 1C**) and HTLV-1 proviral load (p= 0.0141; **Figure 1E**) levels were significantly higher in the symptomatic group.

### 3.3 *TLR3* gene expression, plasma cytokines and HTLV-1 proviral load among individuals with different genotypes of *TLR3* rs5743305 T/A and *TLR3* rs3775291 C/T polymorphisms

**Figure 2:**
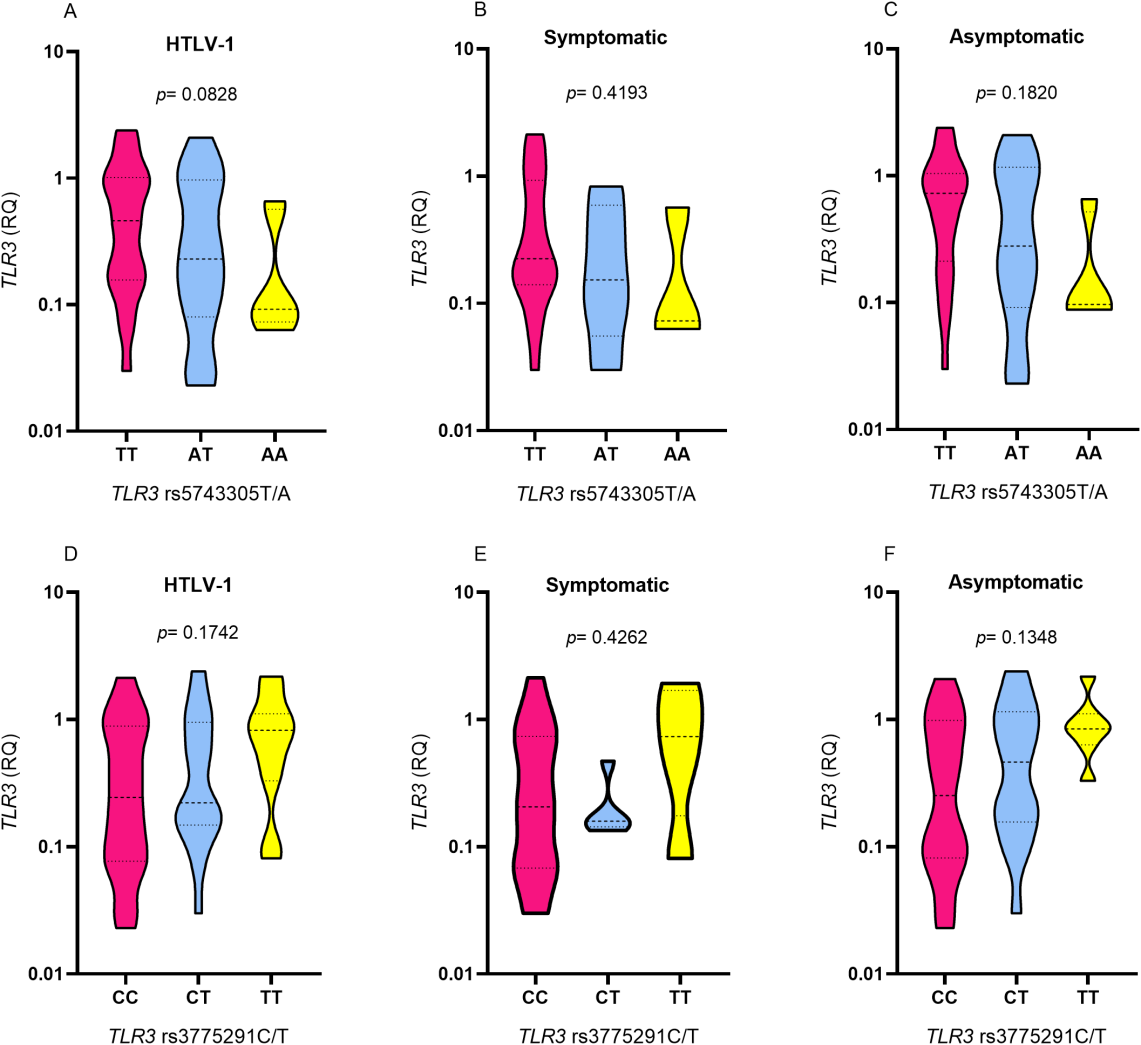
Evaluation of TLR3 gene expression levels among individuals with different genotypes for TLR3 rs5743305 T/A in the group **(A)** with HTLV-1, **(B)** symptomatic and **(C)** asymptomatic; among individuals with different genotypes for TLR3 rs3775291 C/T in the group **(D)** with HTLV-1, **(E)** symptomatic and **(F)** asymptomatic. Kruskal-Wallis test.

*TLR3* expression levels were lower in HTLV-1 individuals carrying the AA polymorphic genotype for the *TLR3* rs5743305 T/A variation in the HTLV-1, symptomatic and asymptomatic groups (**Figures 2A-C**) and higher in carriers of the TT polymorphic genotype for *TLR3* rs3775291 C/T in the HTLV-1, symptomatic and asymptomatic groups (**Figures 2C-F**), but no statistically significant difference was observed, regardless of the presence or absence of symptoms.

**Figure 3:**
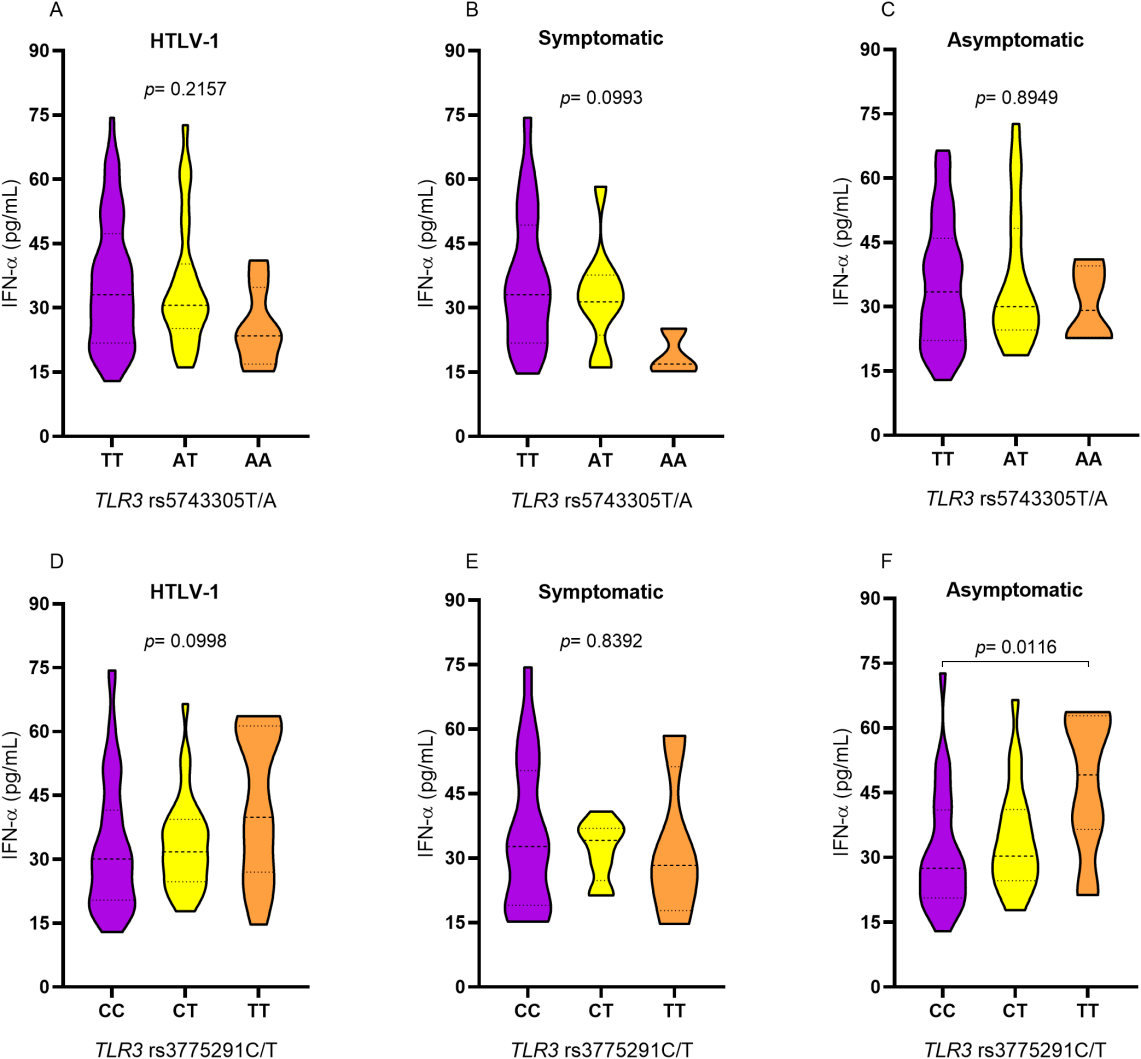
Evaluation of plasma IFN-α levels among individuals with different genotypes for TLR3 rs5743305 T/A in the group **(A)** with HTLV-1, **(B)** symptomatic and **(C)** asymptomatic; among individuals with different genotypes for TLR3 rs3775291 C/T in the group **(D)** with HTLV-1, **(E)** symptomatic and **(F)** asymptomatic. Kruskal-Wallis test.

No significant difference in IFN-α levels was found between *TLR3* rs5743305 T/A genotypes in individuals with HTLV-1 (**Figure 3A**), nor when considering the presence (**Figure 3B**) or absence (**Figure 3C**) of related symptoms. Similarly, no difference in IFN-α concentrations was observed for TLR3 rs3775291 C/T variation in the HTLV group (**Figure 3D**) or the symptomatic group (**Figure 3E**). However, in the asymptomatic group, IFN-α levels were significantly higher in individuals carrying the polymorphic TT genotype compared to those carrying the CC genotype (*p*= 0.0116; **Figure 3F**) for the *TLR3* rs3775291 C/T polymorphism.

**Figure 4:**
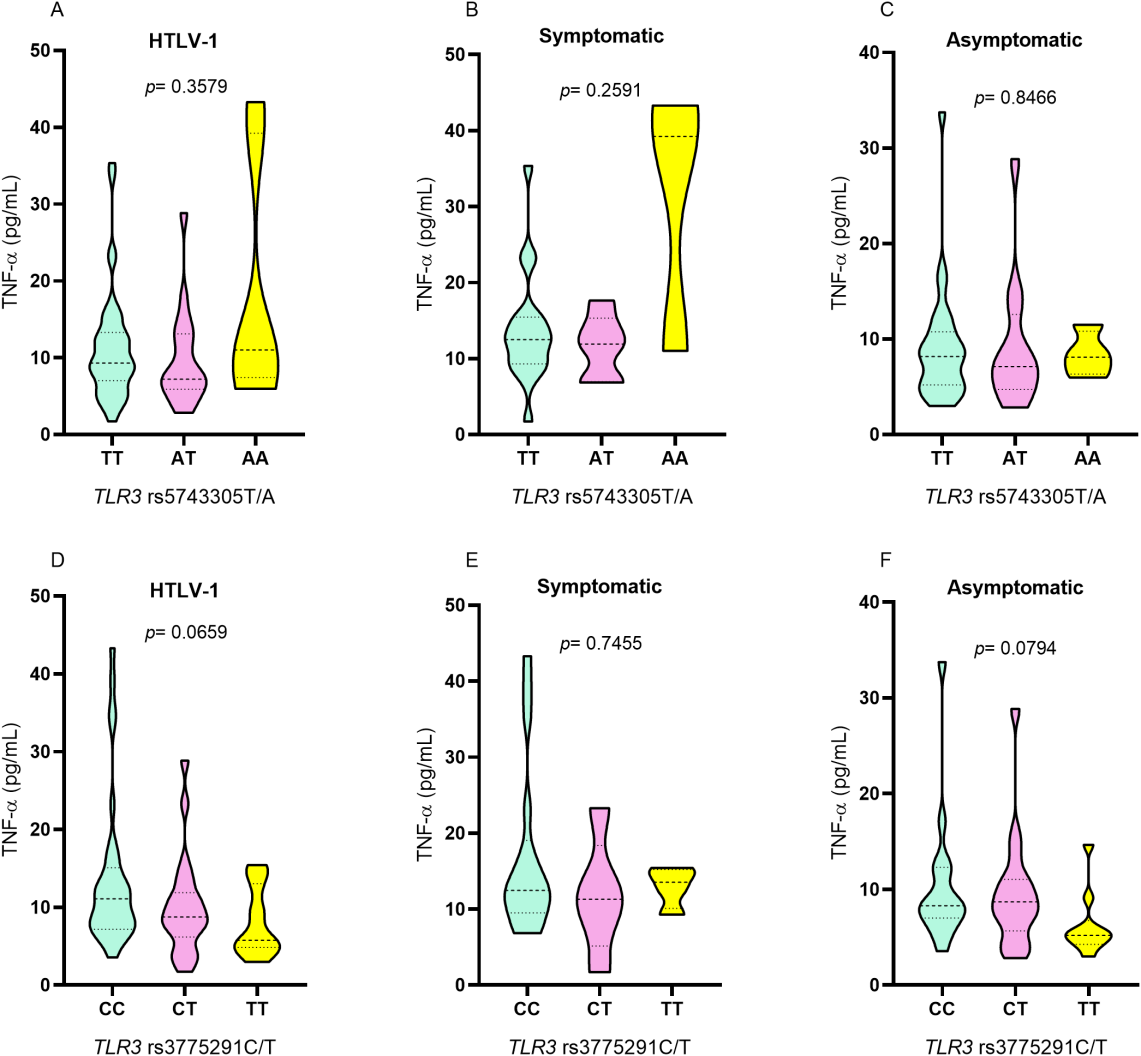
Evaluation of plasma TNF-α levels among individuals with different genotypes for *TLR3* rs5743305 T/A in the group **(A)** with HTLV-1, **(B)** symptomatic and **(C)** asymptomatic; among individuals with different genotypes for *TLR3* rs3775291 C/T in the group **(D)** with HTLV-1, **(E)** symptomatic and **(F)** asymptomatic. Kruskal-Wallis test.

Analyses of TNF-α levels showed no statistical difference between individuals carrying the genotypes for the *TLR3* rs5743305 T/A variation (**Figures 4A-C**). Although carriers of the TT polymorphic genotype for *TLR3* rs3775291 C/T presented lower levels of TNF-α (**Figures 4D, E**), there was no significant difference between individuals carrying the different genotypes for *TLR3* rs3775291 C/T (**Figures 4D-F**).

**Figure 5:**
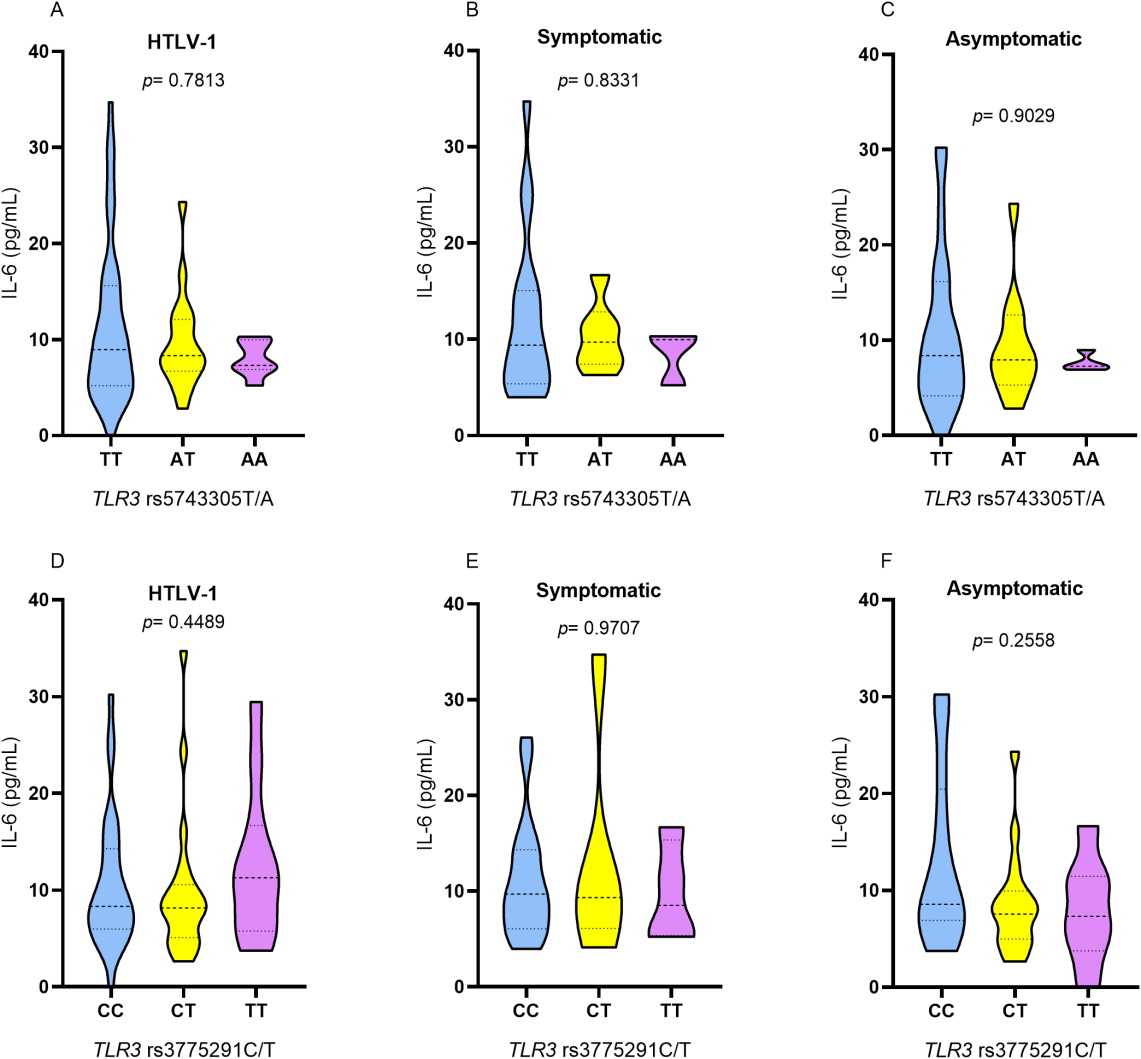
Evaluation of plasma IL-6 levels among individuals with different genotypes for TLR3 rs5743305 T/A in the group **(A)** with HTLV-1, **(B)** symptomatic and **(C)** asymptomatic; among individuals with different genotypes for *TLR3* rs3775291 C/T in the group **(D)** with HTLV-1, **(E)** symptomatic and **(F)** asymptomatic. Kruskal-Wallis test.

IL-6 levels did not show a statistically significant difference between individuals carrying the different genotypes for TLR3 rs5743305 T/A and TLR3 rs3775291 C/T in the HTLV-1, symptomatic, and asymptomatic groups (**Figures 5A-F**).

**Figure 6:**
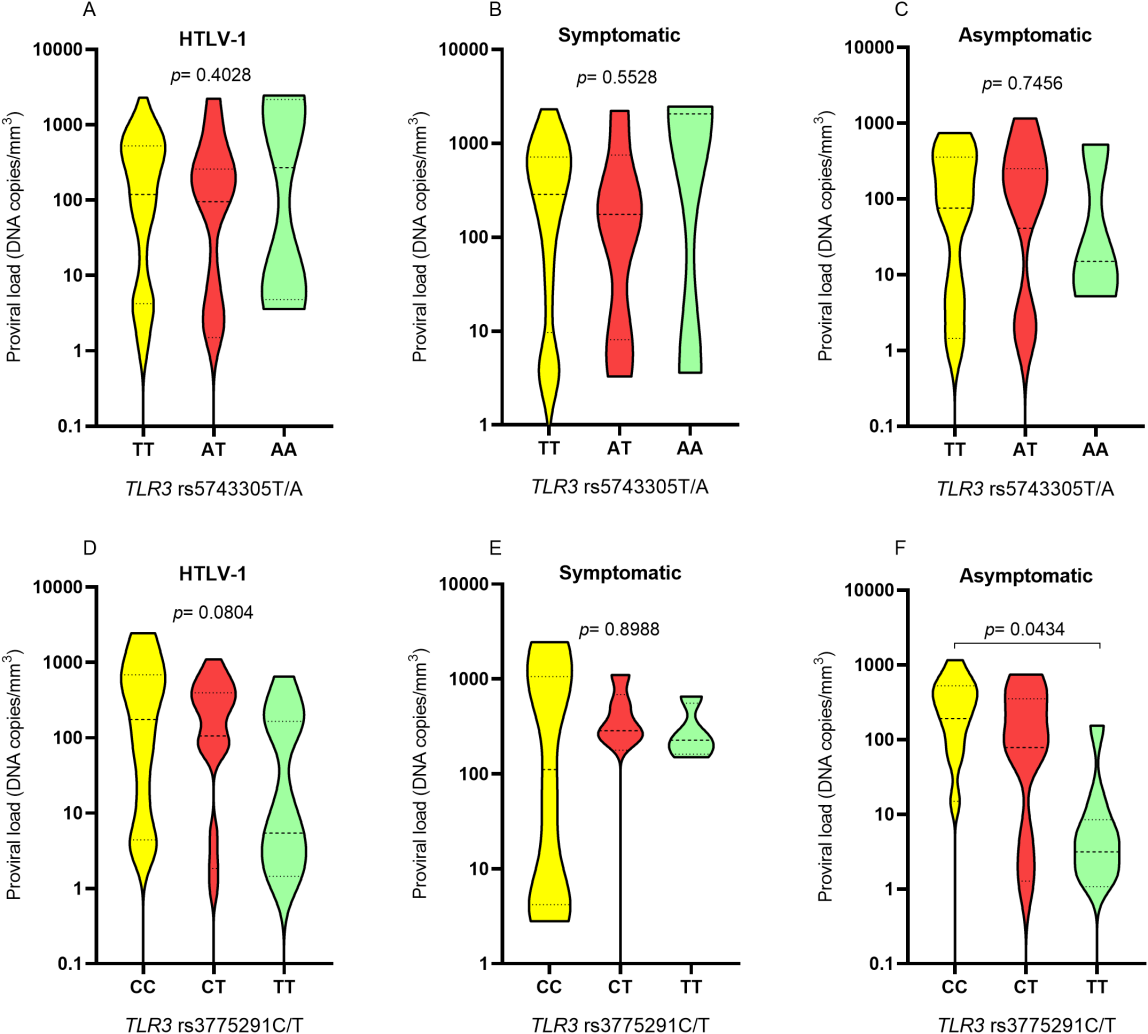
Assessment of proviral load levels among individuals carrying different genotypes for *TLR3* rs5743305 T/A in the group **(A)** with HTLV-1, **(B)** symptomatic and **(C)** asymptomatic; among individuals carrying different genotypes for *TLR3* rs3775291 C/T in the group **(D)** with HTLV-1, **(E)** symptomatic and **(F)** asymptomatic. Kruskal-Wallis test.

The concentration of HTLV-1 proviral load was not different between individuals carrying the different genotypes for *TLR3* rs5743305 T/A in the HTLV-1, symptomatic and asymptomatic groups (**Figures 6A-C**). Regarding the *TLR3* rs3775291 C/T variation, there was no difference in the viral load concentration between the genotypes in the HTLV-1 (**Figure 6D**) and symptomatic (**Figure 6E**) groups. However, in the asymptomatic group, carriers of the polymorphic TT genotype had a lower proviral load than those with the CC genotype (*p*= 0.0434; **Figure 6F**) for *TLR3* rs3775291 C/T.

Expression levels of *TLR3*, IFN-α, TNF-α, IL-6, and proviral load were compared across different genotypes of the *TLR3* rs5743305 T/A and *TLR3* rs3775291 C/T polymorphisms, categorized by sex. No significant differences were found in these analyses (see **Supplementary Figures 1**, **2**).

### 3.4 Multiple evaluations of variables

In the multiple logistic regression analysis, an association was observed between the presence of symptoms and an increase in proviral load (*p*= 0.0287) and plasma levels of TNF-α (*p*= 0.0351) (**Table 3**). Multiple linear regression analysis assessed the association of IFN-α levels with variables among all HTLV-1-infected individuals, including both symptomatic and asymptomatic. In the group with HTLV-1 and symptomatic, no association was observed between any of the variables investigated and IFN-α levels. In the group of asymptomatic individuals, an association was observed between IFN-α levels and TNF-α (β: 1.091; 95% CI: 0.150 – 2.031; *p*= 0.0225) and IFN-α levels with the TT polymorphic genotype for *TLR3* rs3775291 C/T (β: 25.537; 95% CI: 11.611 – 39.463; *p*= 0.0035) (**Table 4**).

**Table 3:** Multiple logistic regression of variables associated with individuals with symptoms of HTLV-1-related diseases.

Variables	*OR*	CI 95%	*p*
Symptoms			
Proviral load	1.002	1.000 – 1.003	0.02875
TNF-α	1.104	1.007 – 1.211	0.03511

**Table 4:** Multiple linear regression analysis of plasma IFN-α levels in relation to the variables investigated in the group of people living with HTLV-1 and according to the presence and absence of symptoms.

IFN-α			
Variables	Estimate	CI 95%	*P*
HTLV-1			
Proviral load	0.001	-0.010 – 0.013	0.8325
*TLR3* expression	10.391	-3.448 – 24.231	0.1310
TNF-α	0.354	-0.529 – 1.237	0.4081
IL-6	0.365	-0.634 1.364	0.4500
*TLR3* rs5743305 T/A			
TT	Ref		
AT	-1.062	-18.937 – 16.812	0.9013
AA	-14.975	-44.974 – 15.023	0.3057
*TLR3* rs3775291 C/T			
CC	Ref		
CT	-2.849	-20.777 – 15.078	0.7405
TT	-6.274	-25.884 – 13.335	0.5073
Symptomatic			
Proviral load	0.001	-0.010 – 0.013	0.8325
*TLR3* expression	10.391	-3.448 – 24.231	0.1310
TNF-α	0.354	-0.529 – 1.237	0.4081
IL-6	0.365	-0.634 – 1.364	0.4500
*TLR3* rs5743305 T/A			
TT	Ref		
AT	-1.062	-20.777 – 15.078	0.9013
AA	-14.975	-25.884 – 13.335	0.3057
*TLR3* rs3775291 C/T			
CC	Ref		
CT	-2.849	-44.974 – 15.023	0.7405
TT	-6.274	-18.937 – 16.812	0.5073
Asymptomatic			
Proviral load	0.019	-0.008 – 0.040	0.1624
*TLR3* expression	2.934	-5.421 – 11.188	0.4598
TNF-α	1.137	0.150 – 2.031	0. 0217
IL-6	0.176	-0.806 – 0.723	0.6885
*TLR3* rs5743305 T/A			
TT	Ref		
AT	1.206	-10.532 – 11.209	0.8255
AA	-4.699	-21.047 – 10.214	0.5406
*TLR3* rs3775291 C/T			
CC	Ref		
CT	3.404	-8.414 – 13.236	0.5492
TT	27.612	11.611 – 39.463	0.0035

**Figure 7:**
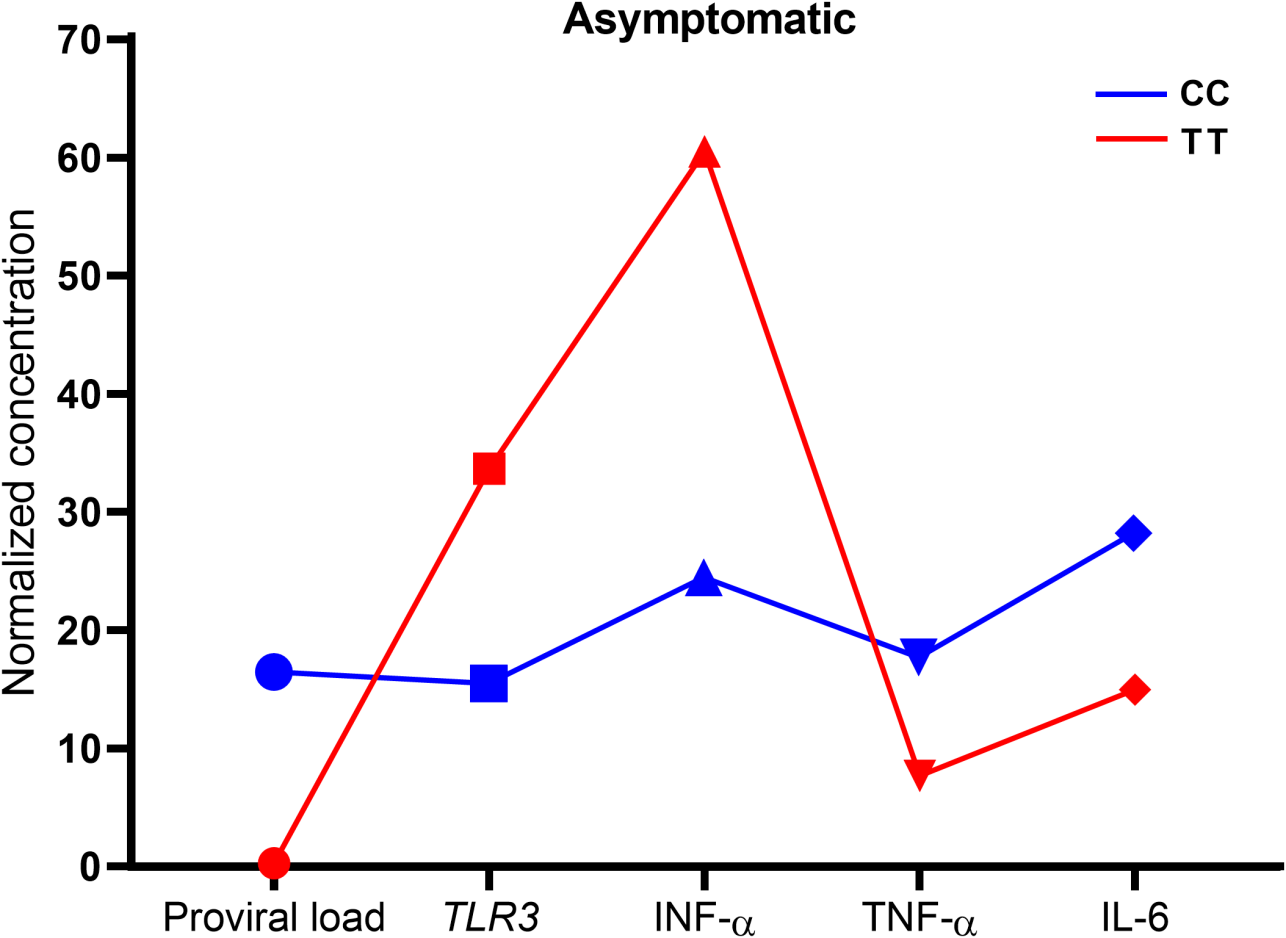
Assessment of the profile of the investigated variables of carriers of the wild (CC) and polymorphic (TT) genotype for the TLR3 rs3775291 C/T variation in the group of asymptomatic individuals.

Based on the results observed in the group of asymptomatic individuals, the values of all investigated variables were normalized to construct and evaluate a profile of carriers of the wild genotype (CC) and the polymorphic genotype (TT) for the *TLR3* rs3775291 C/T variation. The profile of carriers of the polymorphism was characterized by elevated levels of *TLR3* and IFN-α and lower levels of proviral load, TNF-α and IL-6 compared to the profile of those with the wild-type genotype (**Figure 7**).

## 4 Discussion

TLR3 recognizes dsRNA (Kawai and Akira, 2010), which is characterized by being an intermediate product during the replication and transcription of most viruses (Alexopoulou et al., 2001; Rathinam and Fitzgerald, 2011). Upon recognizing dsRNA, the production of type I IFN and pro-inflammatory cytokines is induced (Takeda and Akira, 2005; Blasius and Beutler, 2010). The mechanisms by which TLR3 recognizes HTLV-1 are not yet fully understood. Studies suggest that when the retroviral genome dimerizes and forms a secondary structure, a double-stranded RNA is possibly generated, which can be recognized by TLR3 (Russell et al., 2004; Greatorex, 2004; Watts et al., 2009). HTLV-1 generates several forms of nucleic acid during its replication cycle, such as RNA, RNA/DNA intermediates, ssDNA, and dsDNA. This allows different immunological sensors to detect the presence of the virus and activate immune responses, including TLR3 (Van montfoort et al., 2014; Mohanty and Harhaj, 2023).

HTLV-1 infection status can be affected by various immunological factors and the genetic variability of the host (Yamauchi et al., 2021). The onset of the diseases associated with the HTLV-1 infection can be complexly affected by these factors. Therefore, the present study investigated the association of polymorphisms in the *TLR3* gene with susceptibility to HTLV-1 infection, as well as its influence on the development of symptoms of inflammatory diseases associated with the virus and on the levels of inflammatory and antiviral cytokines, seeking to identify possible factors that may affect the heterogeneity of the immune response.

This study represents the first evaluation of the *TLR3* rs5743305 T/A polymorphism concerning HTLV-1 infection. The genotypic and allelic frequencies between individuals with HTLV-1 and the control group showed no significant differences, indicating that this polymorphism is likely unrelated to HTLV-1 infection susceptibility.

Some studies have found an association between the *TLR3* rs5743305 T/A polymorphism and viral infections. In HBV infection, the AA for the *TLR3* rs5743305 T/A variation was more frequent in the group with chronic hepatitis B compared to the control group. Furthermore, the A allele was associated with a decrease in spontaneous HBsAg seroclearance in Caucasian patients, suggesting that it may represent an important factor in the immunological control of HBV infection (Fischer et al., 2018). In contrast, in tick-borne encephalitis viral infection, the TT genotype for the *TLR3* rs5743305 T/A variant was more frequent in the infected group compared to the control, which was considered a risk genotype (Grygorczuk et al., 2017).

Variations in study results may be attributed to factors such as ethnic diversity among the populations examined, which can affect the prevalence of polymorphisms and their impact on disease progression. The studies that identified an association between the *TLR3* rs5743305 T/A polymorphism and viral infections largely involved Caucasian populations (Grygorczuk et al., 2017; Fischer et al., 2018) and, consequently, genetically more homogeneous, while studies carried out in different population groups presented divergent results, as observed in the present study that evaluated a mixed-race population. The study assessed a population from the Brazilian Amazon, which has genetic contributions from Europeans, indigenous peoples, and Africans, resulting in notable genetic diversity (Santos et al., 2010).

Evaluation of the *TLR3* rs3775291 C/T polymorphism showed that the genotypic and allelic frequencies did not differ between the HTLV-1 and control groups, suggesting that the polymorphism possibly does not influence susceptibility to HTLV-1 infection. In the context of dengue virus infection, research has suggested that the presence of the polymorphic TT genotype for *TLR3* rs3775291 C/T was observed with greater frequency in the infected cohort compared to the control group (Singh et al., 2021). This genotype has been identified as a risk factor for susceptibility to dengue virus infection. The CT genotype for the *TLR3* rs3775291 C/T variant was more frequent in the group of patients with an advanced stage of HIV-1 infection when compared to the control group, which may influence the risk of infection progression (Singh and Samani, 2022). In HBV infection, Rong et al. (2013) and Fischer et al. (2018) reported that the polymorphic T allele for the *TLR3* rs3775291 C/T variation increased the risk of HBV infection. In contrast, Mickienė et al. (2002) evaluated the *TLR3* rs3775291 C/T polymorphism in tick-borne viral encephalitis and identified a lower frequency of the *TLR3* rs3775291 C/T polymorphic genotype TT in infected individuals. The frequency of the polymorphism in the infected group was nearly half that observed in the healthy group, indicating that it may serve as a protective factor against infection.

These contradictory studies on the role of *TLR3* rs3775291 C/T genetic variation in susceptibility to viral infection show that the mechanism by which *TLR3* rs3775291 C/T affects susceptibility/resistance to viral infections is not yet fully understood. The different results observed in these studies may suggest that the effect of the *TLR3* rs3775291 C/T polymorphism may depend on the type of virus causing the infection, since some viruses have different pathogenic components.

Comparison of allelic and genotypic frequencies of the *TLR3* rs5743305 T/A polymorphism between groups with and without symptoms of HTLV-1-associated diseases showed no statistically significant difference. Polymorphic variants for *TLR3* rs5743305 T/A were associated with a low humoral and cellular response to measles vaccination, presenting lower levels of measles-specific antibodies and showing a lower lymphoproliferative response compared to wild type (Dhiman et al., 2008). In HBV infection, no association was observed between the polymorphism and the development of hepatocellular carcinoma in patients with the viral infection (Li and Zheng, 2013). These data suggest that in HTLV-1 infection, the *TLR3* rs5743305 T/A polymorphism does not appear to alter *TLR3* expression and does not influence the HTLV-1 infection status, since the polymorphism was not associated with the presence of clinically established diseases related to the virus.

In our study, bivariate analysis showed that the frequency of the *TLR3* rs3775291 C/T polymorphism was higher in the group of asymptomatic individuals compared to individuals with the presence of symptoms of HTLV-1-associated diseases. Nevertheless, an analysis using multiple logistic regression of the variables examined in relation to HTLV-1-associated disease symptoms indicated that the *TLR3* rs3775291 C/T polymorphism was not linked to clinical manifestations resulting from viral infection. The results suggest a probable influence of the *TLR3* rs3775291 C/T polymorphism on the HTLV-1 infection status, possibly associated with a lower chance of developing symptoms, but it is not a direct predictor of clinical manifestations when other variables are considered.

The *TLR3* rs3775291 C/T polymorphism affects the protein’s ability to bind dsRNA, resulting in a reduced signaling cascade compared to the wild-type form (Ranjith-Kumar et al., 2007; Zhou et al., 2011). Although this functional alteration may impact the activation of TLR3-mediated immune responses, influencing the intensity of the inflammatory response during viral infections, it did not demonstrate a direct relationship with the manifestation of various symptoms of diseases associated with HTLV-1. This suggests that other viral and immunological factors may have more relevant roles in the development of HTLV-1-associated diseases, such as proviral load and the inflammatory cytokine TNF-α (Starling et al., 2013; Neco et al., 2017).

Although the association of the *TLR3* rs3775291 C/T polymorphism with the presence of symptoms or clinical outcomes has been identified in other studies evaluating different types of infections, none of these studies performed a multiple analysis that included the immunological variables involved in TLR3 signaling. For example, polymorphism was associated with protection against HIV-1 infection (Sironi et al., 2012), with lower viremia in cytomegalovirus infection (Redondo et al., 2022) and with a lower chance of developing hepatocellular carcinoma in individuals younger than 40 years of age infected with HBV (Chen et al., 2017). The lack of results from analyses of the *TLR3* rs3775291 C/T polymorphism in relation to multiple immunological factors resulting from TLR3 signaling makes it difficult to compare with our findings resulting from multiple analyses, which are considered a more detailed evaluation.

The comparison of cytokine levels between patients with and without symptoms of HTLV-1-associated diseases indicated that individuals with symptoms had higher levels of TNF-α. The results found corroborate other studies that report that the increase in the frequency of cells expressing TNF-α is directly associated with the pathogenesis of HTLV-1 infection, contributing to the exacerbated inflammatory environment observed in conditions such as HAM (Santos et al., 2004; Nakamura et al., 1993; Neco et al., 2017). In the cerebrospinal fluid (CSF) of patients with MAH, it is possible to find elevated levels of IFN-γ and TNF-α (Quaresma et al., 2015). The most widely accepted hypothesis for the development of HAM is that neuronal damage occurs due to inflammation in the central nervous system (CNS), triggered by cytokines secreted by infected CD4^+^ T cells. The main cytokines involved in this process are IFN-γ and TNF-α, which contribute to the neurodegeneration associated with the disease (Osame, 2002; Araujo and Silva, 2006).

The assessment of HTLV-1 proviral load levels indicated that individuals presenting with symptoms exhibited higher levels compared to those without symptoms associated with inflammatory diseases. Multiple logistic regression analysis confirmed that higher proviral loads were linked to clinical symptoms of HTLV-1 infection. This suggests that proviral load is a robust marker that is independent of the presence of other immunological and genetic factors. High levels of proviral load may be directly linked to the risk of developing inflammatory diseases, probably due to active viral replication and persistent inflammatory response (Grassi et al., 2011; Olindo et al., 2006).

In our study, *TLR3* gene expression levels did not show significant differences between individuals with different genotypes for the *TLR3* rs5743305 T/A and *TLR3* rs3775291 C/T polymorphisms. In cases of HCV infection, *TLR3* expression levels were also unaffected by the *TLR3* rs5743305 T/A polymorphism. However, the *TLR3* rs3775291 C/T polymorphism was linked to higher *TLR3* expression levels and the absence of infection by the HCV1a viral genotype, which is associated with severe prognosis (Askar et al., 2009). These findings indicate that, in certain viral infections, the *TLR3* rs3775291 C/T polymorphism may be related to increased *TLR3* levels and the progression of these infections.

In multiple linear regression analysis, higher IFN-α levels were associated with the rs3775291 C/T polymorphism. Since this polymorphism promotes amino acid changes in the protein (Leu412Fen), it may likely influence the functionality of the *TLR3* receptor, promoting a more efficient activation of the signaling pathway and, consequently, inducing greater IFN-α production. IFN-α induces control of different types of viral infections (Levy et al., 2011). This cytokine acts by repressing the HTLV-1 viral cycle during infection (Cachat et al., 2013). Furthermore, the use of IFN-α as therapy in patients with HAM has been shown to reduce the percentage of inflammatory cells in the cerebrospinal fluid and blood, contributing to the clinical improvement of the patient (Feng et al., 2004). Therefore, the *TLR3* rs3775291/T polymorphism may potentially enhance the efficiency of the antiviral response and mitigate the progression of HTLV-1 infection in asymptomatic individuals.

There are limited studies in literature examining the genetic variations *TLR3* rs5743305 T/A and *TLR3* rs3775291 C/T in relation to IFN-α levels. Our study is one of the first to perform this evaluation, which makes it difficult to establish the role of these polymorphisms in relation to cytokine levels in viral infections. IFN-I induces the expression of several IFN-stimulated genes (ISGs) in host cells, which act at different stages of viral replication, inhibiting the replication and transcription processes of different viruses (Schoggins et al., 2011; Hoffmann et al., 2015). Thus, the production of more adequate levels of the cytokine could aid in the immunological control of HTLV-1 infection, influencing the maintenance of the asymptomatic state of the infection.

While the study provides valuable insights into the HTLV-1 infection status, it is limited by the inability to assess more specific components of the TLR3 signaling pathway, such as IP-10/CXCL10 and ISGs. Changes in the synthesis of IP-10/CXCL10 are known to affect the inflammatory response (Harvey et al., 2003; Zhang et al., 2014). Therefore, identifying ISGs may help determine differences in antiviral response among individuals with or without the TLR3 rs3775291 C/T polymorphism, particularly among asymptomatic individuals.

Since the *TLR3* rs3775291 C/T polymorphism has been associated with the severity of COVID-19, mainly in men, influencing the autophagy process and affecting TNF production and susceptibility to SARS-CoV-2 infection (Croci et al., 2022), we evaluated the influence of the *TLR3* rs5743305 T/A and rs3775291 C/T polymorphisms on HTLV-1 infection according to sex. However, there was no significant difference in the levels of *TLR3* expression, cytokines, and proviral load in the male and female groups. HTLV-1 infection is more common in women, likely because men transmit the infection more effectively to women. The frequency of transmission of HTLV-1 infection from men to women is approximately 60%, while the frequency of transmission from women to men is 0.4% (Kajiyama et al., 1986; Paiva and Casseb, 2014). Therefore, it is probable that the epidemiological characteristics of HTLV-1 transmission have impacted the analyses with respect to sex.

It is important to highlight that among the studies carried out on polymorphisms in the *TLR3* gene, none performed analyses of the *TLR3* rs5743305 T/A and *TLR3* rs3775291 C/T polymorphisms together with the quantification of the proviral load, antiviral and inflammatory cytokines, which were evaluated in the present study, which allowed a more consistent analysis of these polymorphisms in HTLV-1 infection status. The identification of polymorphisms that influence the HTLV-1 infection status can assist in personalized medicine, through studies of personalized approaches in clinical management, helping to predict which individuals are at greater risk of developing complications. However, we consider a more comprehensive investigation necessary, through analyses with larger population samples, from different ethnicities, and the inclusion of other polymorphisms in the *TLR3* gene to provide a greater understanding of the real impact of the genetic variations studied in the context of HTLV-1 infection.

## 5 Conclusion

In summary, the polymorphisms were not linked to susceptibility to HTLV-1 infection or the infection status. Disease symptoms correlated with increased TNF-α levels and proviral load. The *TLR3* rs5743305 T/A polymorphism was not connected with changes in *TLR3* and IFN-α levels. For the *TLR3* rs3775291 C/T polymorphism, asymptomatic individuals with the TT polymorphic genotype exhibited significantly higher IFN-α levels and lower proviral load. Asymptomatic individuals carrying the TT polymorphic genotype for *TLR3* rs3775291 C/T demonstrated elevated levels of *TLR3* and IFN-α, and reduced levels of proviral load, TNF-α, and IL-6 compared to those with the wild-type genotype. Although polymorphisms in the *TLR3* gene have not been associated with the presence of symptoms related to HTLV-1 inflammatory diseases, the *TLR3* rs3775291 C/T polymorphism seems to contribute to improved control of the infection and inflammatory process among asymptomatic individuals.

## Data Availability

The original contributions presented in the study are included in the article/**Supplementary Material**. Further inquiries can be directed to the corresponding author.
