# Supplementary material for: TLR3 rs3775291 C/T polymorphism is associated with elevated IFN-α level in asymptomatic HTLV-1 infection
Source: Front Cell Infect Microbiol. 2025 Aug 4;15:1604259. doi: 10.3389/fcimb.2025.1604259 (PMC12358490; doi:10.3389/fcimb.2025.1604259)
Supplement: Supplementary file 1 [file DataSheet1.docx]

**Supplementary Material**


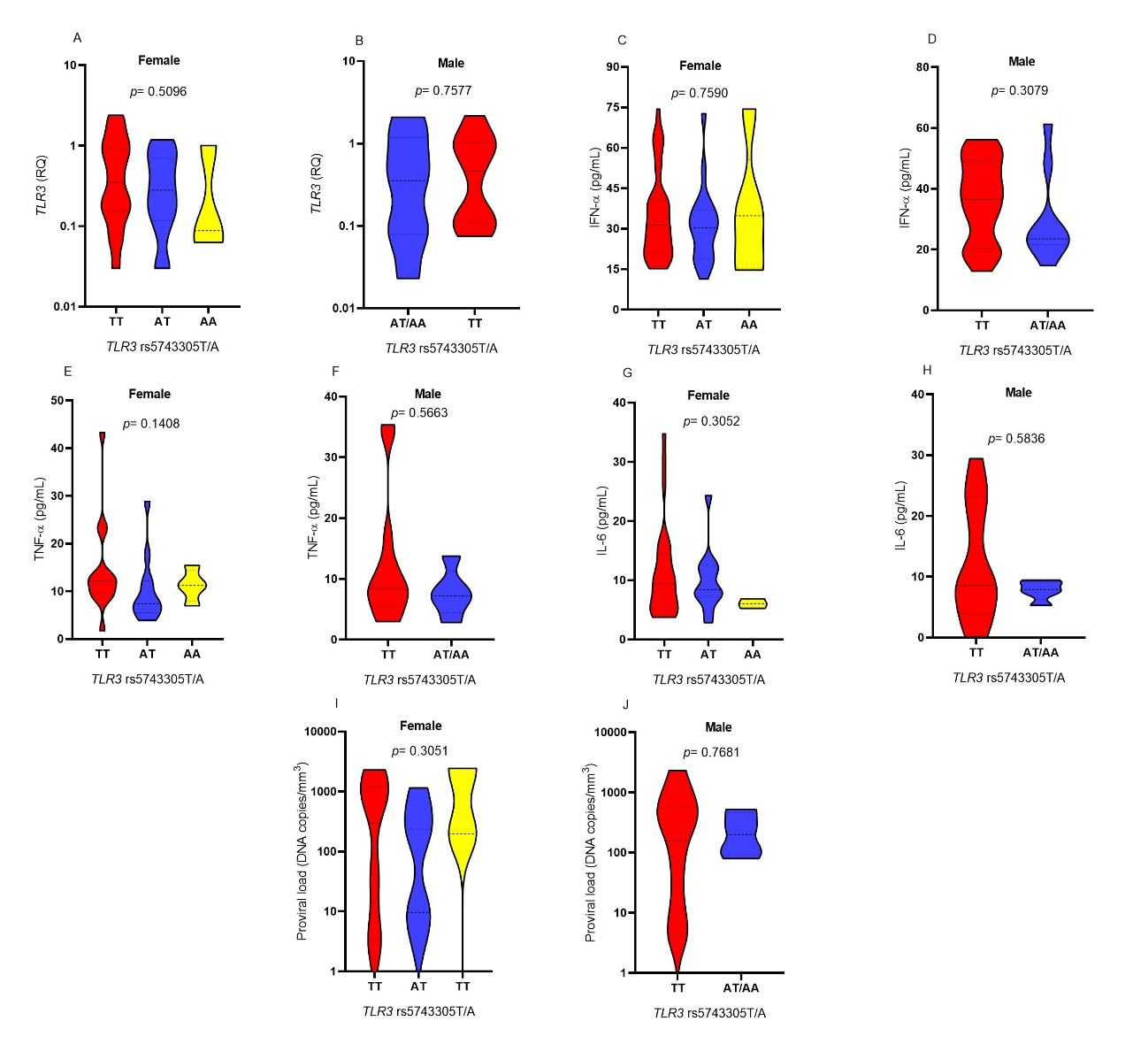


**Supplementary Figure 1** – Assessment of the levels of (**A** and **B**) gene expression of TLR3, (**C** and **D**) IFN-α, (**E** and **F**) TNF-α, (**G** and **H**) IL-6 and (**I** and **J**) proviral load among individuals carrying different genotypes for the *TLR3* rs5743305T/A polymorphism, according to sex.


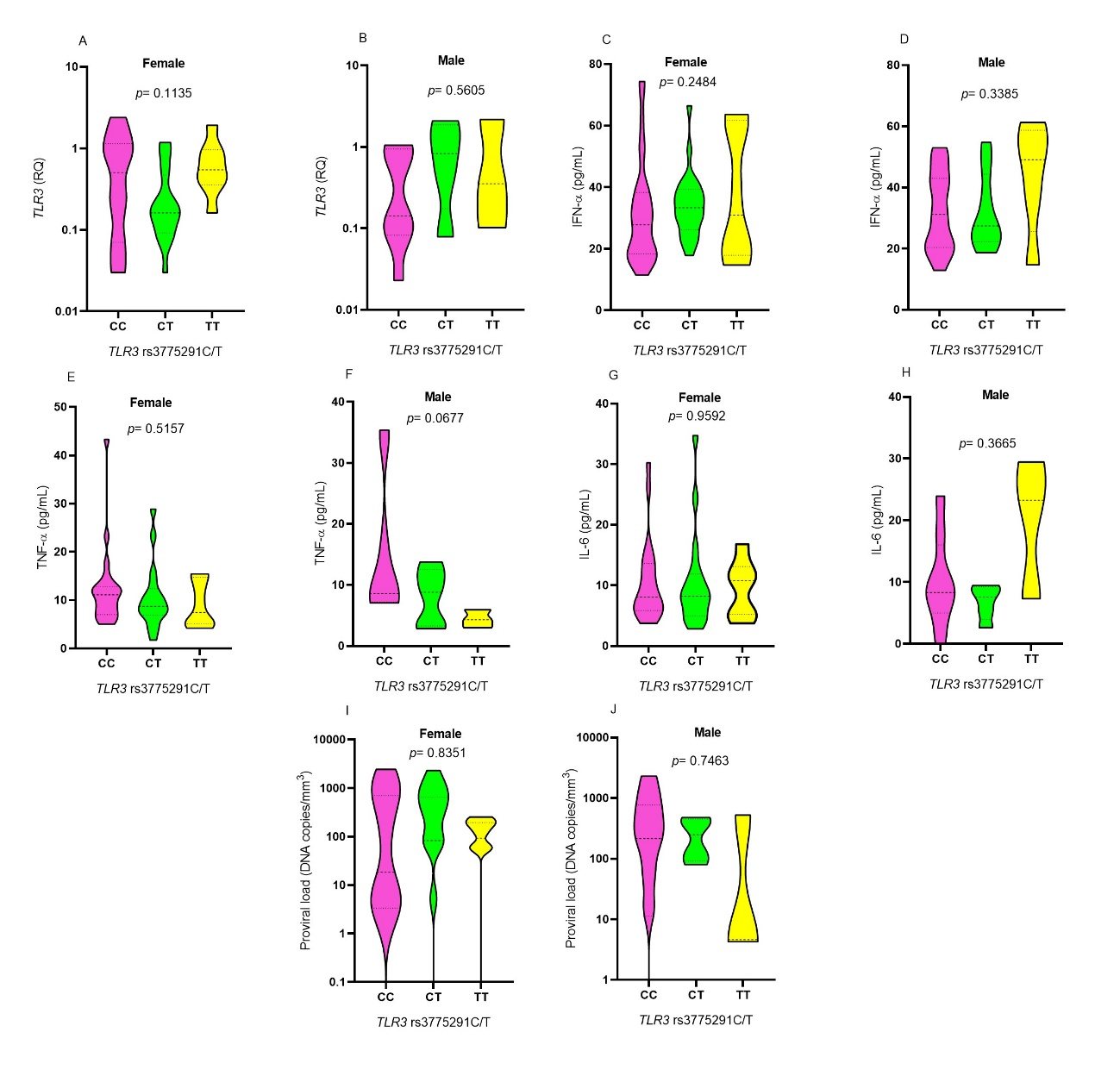


**Supplementary Figure 1** – Assessment of the levels of (**A** and **B**) gene expression of TLR3, (**C** and **D**) IFN-α, (**E** and **F**) TNF-α, (**G** and **H**) IL-6 and (**I** and **J**) proviral load among individuals carrying different genotypes for the *TLR3* rs3775291C/T polymorphism, according to sex.
